# Intra-abdominal pressure monitoring in cardiac surgery: is this the canary in the coalmine for kidney injury?

**DOI:** 10.1007/s10877-022-00933-y

**Published:** 2022-12-22

**Authors:** Wojciech Dabrowski, Philippe Rola, Manu L. N. G. Malbrain

**Affiliations:** 1 First Department of Anaesthesiology and Intensive Therapy, Medical University of Lublin, Lublin, Poland; 2 Intensive Care Unit, Santa Cabrini Hospital, CEMTL, Montreal, Canada; 3 Medical Data Management, Medaman, Geel, Belgium; 4 International Fluid Academy, Lovenjoel, Belgium

## IAP and the kidneys, an inseparable couple

There has been increased awareness about elevated intra-abdominal pressure (IAP) and particularly intra-abdominal hypertension (IAH) and abdominal compartment syndrome (ACS), which can occur with markedly elevated IAP (> 20 mmHg) [1], and that is associated with significant morbidity and mortality [2]. Increased IAP impacts each organ system within and far outside the abdominal cavity. The kidneys have been considered the canary in the coal mine for IAH, with oliguria as the usual first sign of acute kidney injury (AKI) [3]. Mean perfusion pressure (MPP) is the difference between mean arterial pressure (MAP) and central venous pressure (CVP) and has been associated with the progression of organ system injury [4, 5]. A more specific marker for resistive abdominal forces may be abdominal perfusion pressure (APP), calculated as the difference between MAP and IAP [6]. And more specifically, the filtration gradient (FG), calculated as the difference between MAP and twice the IAP, has been suggested to assess glomerular filtration and correlated moderately with renal blood flow and microcirculatory perfusion, whereas APP did not [7]. Increased renal vascular resistance with elevated IAP might account for this [7]. This warrants appropriate IAP monitoring, primarily done using homemade or commercial pressure measurements via the bladder catheter in an intermittent fashion [8]. As conventional bladder pressure monitoring requires the transient obstruction of the catheter, continuous monitoring of IAP could not be performed in the past and required human intervention (e.g. via the use of a 3-way Foley catheter with continuous irrigation). In this issue of the journal, Khanna and colleagues, describe a new monitoring technique that additionally allowed for assessing both cumulative (pressure time burden) and continuous (assessment of the effect of treatment) aspects of IAP, which had never previously been done via the bladder [9].

## What does the study tell us?

First of all, we would like to thank and congratulate the authors on this study, which, in our opinion, takes us one step further in the monitoring realm of acutely ill patients [9]. Using a novel technology that requires no further invasiveness than the insertion of a dedicated Foley catheter (the Accuryn Monitoring System, Potrero Medical, Hayward, CA, USA), the authors monitored IAP continuously for 48 h in a cohort of postoperative cardiac surgery patients.

Interestingly, the authors described the presence of significant IAP elevation (IAH > 12 mmHg for > 12 h) in 93% of the patients, making it essentially a feature of the post-cardiac surgery course, which previously remained unnoticed. More importantly, the authors also presented a graph illustrating the relation between elevated IAP and decreased urine output, a key parameter in detecting and defining AKI. It would be interesting to see if a pressure–time integral, or area-under-the-curve (AUC) concept applies to splanchnic organ dysfunction and whether continuous abdominal perfusion pressure measurement can play a major prognostic role.

## Intra-abdominal pressure and the kidneys: the relationship works both ways!

The effect of pressure dysregulation on renal function is established, both from the venous backpressure—measured either by CVP [10], or Doppler indices [11, 12] and IAP [13, 14]. Both of these (CVP and IAP) reflect the importance of considering organ perfusion pressure (taking into account the back pressure at the venous side) instead of focusing only on inflow pressure. In a sense, while elevated IAP may be a warning signal for AKI, the reverse may also hold true, that decreasing urine output (oliguria) may act as the canary in the coal mine for IAH and anuria for impending ACS. The ability to monitor both closely and accurately would bring, in our opinion, a potentially important safeguard against both.

**Fig. 1:**
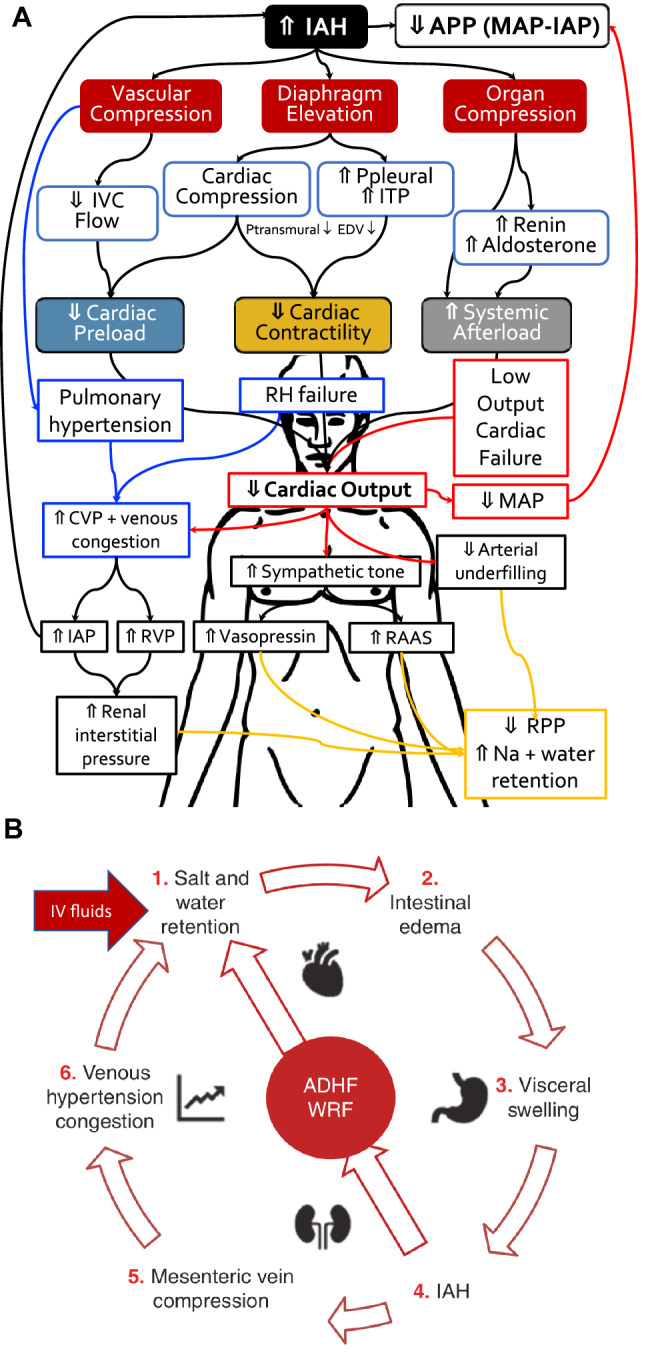
Panel **A** Pathophysiological effect of intra-abdominal hypertension and heart failure (RED arrows indicate forward failure) related venous congestion (BLUE arrows indicate backward failure) on organ function and net effects on salt and water homeostasis (ORANGE arrows). Adapted from Minini et al. with permission [15]. *APP* abdominal perfusion pressure, *CVP *central venous pressure, *IAP* intra-abdominal pressure, *ITP* intrathoracic pressure, *IVC* inferior vena cava pressure *MAP* mean arterial pressure, *RAAS* renin angiotensin aldosterone system, *RH* right heart, *RPP* renal perfusion pressure. Panel **B** The pathophysiological vicious cycle of fluid overload leading to cardio-abdominal-renal syndrome (CARS). Adapted from Minini et al. with permission [15]. *ADHF* advanced decompensated heart failure, *WRF* worsening renal function

We suspect that this study should prompt a similar one in patients with advanced decompensated heart failure (ADHF), who have also been found to have elevated IAP frequently [15]. The concept of cardio-abdominal-renal syndrome (CARS) has been proposed, as congestive heart failure can result in an elevated IAP due to several potential mechanisms such as ascites, bowel edema, ileus, and abdominal wall anasarca (Fig. 1, Panel A) [16]. Splanchnic venous congestion is associated with organ dysfunction [11, 12], likely through decreased renal perfusion pressure, which can be further compromised by external capsular pressure from IAP [15]. This pathophysiology would make ADHF patients potentially an important group to benefit from continuous urine output and IAP monitoring, particularly since mortality is significantly higher in the subgroup with elevated IAP and those with worsening renal failure.

## Intra-abdominal pressure increase during cardiac surgery

The present study is not the first to demonstrate elevated IAP in cardiac surgery patients. However, none of the previous work measured IAP continuously as Khanna and colleagues have done, thereby opening the possibility of uncovering an undermeasured and under detected physiological change. Most of the clinical studies showed increased IAP (measured intermittently) during the first 24–48 h after CABG completion [9, 17–30]. This IAP increase was associated with a decrease in APP and correlated with organ dysfunction [17, 18, 20]. Greater increases in IAP were observed in patients with higher BMI or more blood dilution following cardiopulmonary bypass, and the IAP increase was correlated with postoperative fluid balance and CVP [17, 22, 24, 26–28]. Additionally, patients with IAH received higher doses of vasoactive drugs [22]. In all studies the increase in IAP was temporary with IAP returning to the preoperative levels after 24 h in the majority of patients. However, the development of IAH (ranging from 30 to 50%) was associated with several postoperative complications, including AKI (Table 1).

**Table 1:** The most important papers describing the effect of cardiac surgery on intra-abdominal pressure

Author, year of publication [reference]	Type of surgery	Number of studied patients	Risk factors of IAH	IAP baseline (mmHg)	IAP after surgery (mmHg)	Adverse effect of elevated IAP and comments
Czajkowski [17]	CABG	21	Normovolemic hemodilution	9 (7.5–0)	BW < 75 kg: 12 (9.3–14) BW > 75 kg: 17 (17–18.5)	Correlation between IAP and CVP. IAP higher in obesity. Median IAP returns to baseline (BL) of 9 mmHg after 18 h
Dabrowski [18]	CABG	25	Normovolemic hemodilution, cumulative fluid balance	8.1 ± 1.8 9(8–9)	12.2 ± 3.1 12 (10–16)	IAP relates to disorders in venous outflow from the brain, increase in CVP. Median IAP returns to BL of 9 mmHg (8–10) after 18 h
Dabrowski [20]	CABG and aortic Valve surgery	50 BMI < 25(18) BMI25-30 (23) BMI > 30 (9)	Fluid balance, intra-operative blood dilution	BMI < 25: 4.72 ± 1.19 BMI < 30: 6.69 ± 1.78 BMI > 30: 9.11 ± 0.99	11 ± 4.01 11.3 ± 3.4 12.9 ± 2.02	Decrease in abdominal perfusion pressure. Correlation IAP and BMI. Return to IAP 9 ± 2 mmHg, median of 8(6–11) after 18 h in all BMI groups
Dabrowski [36]	CABG	45	Not studied	6.64 ± 1.87	11.17 ± 3.81	Decrease in coronary perfusion pressure (CoPP). Correlation between IAP and CoPP and PCWP. Correlation CoPP and APP. IAP decreased to 9.08 ± 3.93 mmhg after 18 h
Dalfino et al. [22]	CABG and Off-pump cardiac by-pass surgery	69	Fluid balance	8 (IAH in 32%) 6.5 (no IAH)	14 9	IAP correlated with CVP, risk of AKI, prolonged mechanical ventilation, FB, higher doses of vasopressors. Higher IAP when on pump. Median IAP of 13 mmHg after 24 h (IAH) vs 7 (no IAH)
Iyer D. et al. [25]	CABG and Off-pump cardiac by-pass surgery	108	Fluid balance, duration of aorta cross-clamping	IAH in 46% (n = 50)	NA	Prolonged mechanical ventilation, higher doses of vasopressors, lower pH and PaO_2_/FiO_2_ ratio
Smit M. et al. [28]	CABG, CABG + Valve surgery, Thoracic aortic aneurysm	186	BMI	9.1 ± 4.4	IAH in 26.9% (n = 50) ACS in 2.2% (n = 4)	Correlation IAP with W:H ratio, waist circumference and BMI
Mazeffi et al. [24]	CABG, CABG + Valve surgery,	50	Not studied			Increased risk of AKI
Nazer et al. [27]	CABG	50 (25 with BMI > 30)	BMI	BMI > 30: 10.3 ± 3.3 BMI < 30: 8.4 ± 2.4	15.4 ± 1.6 10.6 ± 1.6	Increased risk of AKI, liver dysfunction, prolonged postoperative mechanical ventilation
Kılıç et al. [21]	CABG, CABG + Valve surgery,	100	Age, hypertension, fluid balance, intra-operative blood dilution, duration of cardiopulmonary by-pass	10.1 ± 2.4 (IAH in 49%) 8.1 ± 2.3	12.2 ± 0.7 (IAH) 9.5 ± 1.6 (no IAH)	Increased incidence of atrial fibrillation, higher doses of vasopressors, higher lactate level, lower central venous saturation, AKI. Correlation with CVP. IAP after 24 h 14.7 ± 3.2 mmHg in IAH group
Ramser et al. [29]	CABG, CABG + Valve surgery,	4128	Risk factor for ACS: Perioperative ejection fraction, high Euroscore 2, duration of cardiopulmonary bypass	ACS in 1% (n = 42)	NA	In the 18 surviving patients, fascial closure was achieved in 72% after a median of 9 days. Outcome predictor in ACS: emergency, BMI, ASA, age
Richer-Séguin et al. [30]	CABG, CABG + Valve surgery,	191	BMI	13 [9–15] (n = 191) 9 [7–10] (no IAH) 15 [13–17] (IAH in 55%, n = 105)	13 [10–15]	IAP independently associated with BMI, CVP and mean pulmonary artery pressure IAP measured 2 h after the admission to the postoperative cardiac intensive care unit was 8 [6–11].
Khanna et al. (present study) [9]	Cardiac surgery	137	NA	6.3 [4.0–8.1]	Within 6 h 10.2 [7.7–13.6] (ETT) and 17.2 [14.1–20.7] (postextubation)	IAP first 24 h: 15.9 [13.6–18.7] IAP next 24–48 h 16.6 [14.5–19.1]. 93% (128/137) of patients spent at least 12 h in IAH grade I, 88% (113/128) of those patients in grade I also had grade II, 47% (53/113) of patients with grade II also had grade III, and 13% (7/53) of patients with grade III also had grade IV IAH

Cardiopulmonary bypass can increase endothelial permeability leading to excessive fluid movement to the extravascular space, followed by tissue edema and increases in IAP [31, 32]. Inappropriate fluid administration perioperatively can lead to (intestinal) fluid accumulation, further contributing to IAH (Fig. 1, Panel B). The amount of extravascular water correlates significantly with the level of IAH [33]. Hypotonic priming, especially with cardiopulmonary bypass-related normovolemic hemodilution can exacerbate extravascular water build up [17]. Perioperative fluid administration should therefore be titrated with caution. Moreover, an increase in IAP above 15 mmHg impairs microcirculation including of the kidneys whereas IAP > 25 mmHg causes critical reduction of renal circulation, and these changes corresponded to a decrease in APP in experimental model of IAH [34]. An elevation of IAP to 15 mmHg for 120 min followed by IAP of 30 mmHg for 120 min caused a reduction in global perfusion, especially in the microcirculation of intestinal and ventricular mucosa, pancreas and the kidneys, and slightly increased cerebral perfusion which was associated with increase in intra-cranial pressure (ICP) [31]. Increased ICP with low cerebral perfusion can result from diminished venous return in IAH, which was observed in both cardiac surgery and critically ill patients [18, 35]. Interestingly, every disorder in cerebral circulation corresponded to increased risk of delirium and poor neurological outcome in cardiac surgery patients [19]. Elevated cerebral venous pressure led to cerebral damage as reflected by increased concentration of blood brain-injury biomarkers [36, 37]. Hence, disturbance in venous outflow following IAH after cardiopulmonary bypass can increase the risk of postoperative delirium and other neurological complications, potentially prolonging hospitalization duration after cardiac surgery.

The cephalic shift of the diaphragm in IAH impairs ventilation, both mechanical and spontaneous by reducing lung and chest wall compliance, lung volumes and increasing inspiratory resistance with high peak and plateau airway pressures [38]. An experimental study showed direct transmission of IAP to the thoracic cavity for approximately 50% [39]. The use of positive end-expiratory pressure (PEEP) counteracts the negative effect of IAH, therefore some PEEP is recommended [38, 40]. This was confirmed by Dalfino and colleagues who noted the prolonged duration of mechanical ventilation in patients with IAH [22]. Additionally, an increase in IAP can impair cardiac function leading to electrocardiographic abnormalities and increasing risk of cardiac arrhythmias [41]. Clinical observations showed an incidence of IAH in approximately 50% of patients undergoing elective cardiac surgery, and this increase was associated with a four-fold increase in postoperative atrial fibrillation [21]. Therefore, elevated IAP can be considered a risk factor that predisposes to postoperative complications after cardiac surgery.

## IAH-induced cardiac dysfunction in cardiac surgery patients

Cardiac dysfunction caused by IAH has been well recognized (Fig. 1, Panel A). An experimental induction of IAH to 40 mmHg caused significant reduction of cardiac output and stroke volume and increase in vascular resistance [42]. Significant elevation of IAP also increases blood pressure in the pulmonary circulation and pulmonary capillary wedge pressure (PCWP) in a dose-related fashion [43]. Reduced cardiac output following IAH decreases microcirculatory perfusion in several organs, with the kidney, small bowel and colon mucosa being the most vulnerable [44]. The acute organ hypoperfusion together with massive inflammatory response increase the risk of organ insufficiency and a vicious cycle leading to ACS. Clinical observations showed that ACS developed in approximately 1% of patients after cardiopulmonary bypass, however it was associated with high mortality of 57% [29]. Importantly, the majority of patients with postoperative ACS were undergoing elective CABG surgery. This fact allows speculating that the stunned heart after the rapid changes in the cardiac perfusion following bypass together with cardiovascular depression following IAH can be a significant risk of ACS and poor outcome. Inappropriate fluid administration, especially fluid overload/accumulation and positive perioperative fluid balance was recognized as one of the most important risk factors for IAH and ACS, while restrictive fluid administration, avoidance of hypotonic crystalloids and use of hypertonic saline to control or slightly increase plasma osmolality was recommended to reduce the risk of IAH [45]. Avoiding IAH in the postoperative period hence eliminates a potential risk factor for cardiac dysfunction.

## Limitations of the present study

Khanna et al. admit to several limitations in their study which is essentially pilot data for the nearly 10 times larger registry that is currently being created by the same group as part of an ongoing prospective study. This study does provide a lot of food for future thoughts. First, the sample size was relatively small and the study may have been underpowered to demonstrate causal relations. Second, since this study, a sub-study of an ongoing data registry had limited information on patient demographics, it was merely observational, and no interventions were prescribed upon increased IAP or presence of AKI. Third, the authors presented a graphical decrease in urinary output that was associated with elevated IAP. However, they did not analyze a potential relationship between IAP and length of postoperative mechanical ventilation, the incidence of delirium and the incidence of postoperative cardiac arrhythmias. Fourth, as IAP data is captured continuously it does not take into account potential confounders like patient position, sedation, pain, delirium, non-invasive ventilation, etc. Fifth, baseline IAP values could only be obtained after induction of anaesthesia (and muscle relaxation). Sixth, important data on fluid administration, fluid balance and concomitant medication (eg. diuretics), are missing. Seventh, it remains unclear why such relatively high IAP values were observed in this specific patient population. The median IAP values after 24 h remain elevated above 15 mmHg and are not in line with previous literature results, albeit performed with intermittent IAP. To play the devil’s advocate one could even argue on the importance of IAP if > 90% of patients exhibit IAH and do relatively well. Eight, unfortunately IAP monitoring stops at ICU discharge—but some patients still have high IAP > 20 mmHg—it would have been interesting to see what happened afterwards. Ninth, the authors provide no information on the relation between KDIGO or AKIN criteria with respect to duration of IAH (above 12 mmHg and other grades) and the duration of low urine output (below 0.5 or 0.3 ml/kg/min). In analogy to the cerebral compartment, the pressure–time burden of IAP is probably closely linked to AKI development. Finally, so far, no validation of continuous IAP has been done compared to the gold standard technique, e.g. intermittent bladder pressure measurement using the height of urine column, with patient supine, at end-expiration, without abdominal muscle contractions and zeroed at the level where the midaxillary line crosses the iliac crest. When examining an evidence based monitoring device we must ask ourselves four questions: (1) does the new device perform as well as the traditional gold standard; (2) does the new device offer us new information (new measured or derived parameters e.g. area under the curve, time above a certain threshold, pressure time burden, compliance, etc.; (3) can we guide/adapt our treatment based on this new information and finally (4) and if we do so, will this new parameter drive treatment effect and improve outcomes? The present paper is on the second point. The other questions need to be answered by future validation studies on continuous IAP in different patient populations, with normal and high BMI and with/without mechanical ventilation, following the WSACS recommendations and guidelines for research [46]. Finally, as we come to a point where the WSACS guidelines for IAH need to be updated [1], should we consider new paradigms of IAH grade based on continuous IAP thresholds (including also spontaneously breathing patients) different from the traditional sedated, mechanically ventilated patients, with intermittent IAP thresholds remains an important question. Should we titrate to a MAP or an APP to ensure that we provide an individualized precision medicine-based approach to organ protection is the next most important question that deserves an answer as well.

## Take home messages

Khanna et al. deserve compliments for executing this study, and while the data presented are hypothesis generating, this will certainly open the doors for several follow-up and validation studies that will answer the questions regarding the prognostic power of pressure time burden, continuity, and accuracy of continuous IAP in a broader population of critically ill patients.

In summary, IAP and AKI go hand in hand and the novel continuous IAP monitoring tool presented herein opens a very interesting door into personalized physiological medicine for critically ill and ADHF patients, prompting both observational and interventional studies to determine how management could be altered with this available information.
